# Modeled exposure to tetrachloroethylene-contaminated drinking water and the risk of placenta-related stillbirths: a case-control study from Massachusetts and Rhode Island

**DOI:** 10.1186/s12940-018-0402-1

**Published:** 2018-07-03

**Authors:** Ann Aschengrau, Lisa G. Gallagher, Michael Winter, Lindsey J. Butler, M. Patricia Fabian, Veronica M. Vieira

**Affiliations:** 1 Department of Epidemiology, Boston University School of Public Health, 715 Albany Street, Talbot 3 East, Boston, MA 02118 USA; 2 Biostatistics and Epidemiology Data Analytics Center, Boston University School of Public Health, 85 East Newton Street, M921B, Boston, MA 02118 USA; 3 Department of Environmental Health, Boston University School of Public Health, 715 Albany Street, Talbot 4 West, Boston, MA 02118 USA; 4 University of California, Irvine, Program in Public Health, 653 East Peltason Drive, Irvine, CA 92697 USA

## Abstract

**Background:**

Residents of Massachusetts and Rhode Island were exposed to tetrachloroethylene (PCE)-contaminated drinking water from 1968 through the early 1990s when the solvent was used to apply a vinyl liner to drinking water mains to address taste and odor problems. Few studies have examined the risk of fetal death among women exposed to solvent-contaminated drinking water. Two previous investigations found moderate increases in the risk of stillbirth among highly exposed women; however, these results were based on a small number of cases. The present case-control study was undertaken to examine further this association with a large number of stillbirths.

**Methods:**

Cases were comprised of stillborn infants delivered between 1968 and 1995 to mothers who resided in 28 Massachusetts and Rhode Island cities and towns with some affected water mains (*N* = 296). Cases were included if the cause of death was placental abruption and/or placental insufficiency. Controls were randomly selected live-born infants who were delivered in the same time period and geographic area (*N* = 783). Data on confounding variables were gathered from vital records and questionnaires. PCE exposure was estimated using a leaching and transport model integrated into water system software.

**Results:**

Mothers with any PCE exposure had a 1.7-fold increase in the adjusted odds of placenta-related stillbirth (95% CI: 1.2–2.4). The adjusted odds ratio (OR) increased as a woman’s exposure level increased: in comparison to unexposed mothers, ORs were 1.5 (95% CI: 1.0–2.3) for low exposure (> 0-median), 1.7 (95% CI: 1.1–2.5) for moderate exposure (>median-90th percentile) and 1.9 (95% CI: 1.1–3.2) for high exposure (>90th percentile) (*p* value for trend = 0.02). A similar pattern was observed when PCE exposure was dichotomized at 40 μg/L, the suggested action guideline for remediation (OR = 1.5, 95% CI: 1.1–2.2 and OR = 2.6, 95% CI: 1.4–4.8, respectively, for PCE exposure <=40 μg/L and > 40 μg/L) (p value for trend = .003).

**Conclusions:**

We observed a linear dose-dependent increase in the odds of stillbirth due to placental abruption and placental insufficiency with prenatal exposure to PCE contaminated drinking water. Because PCE remains a common drinking water contaminant, these findings highlight the importance of considering pregnant women when monitoring, regulating and remediating drinking water supplies.

## Background

Tetrachloroethylene (also called perchloroethylene, perc or PCE) is a solvent commonly used in textile processing, metal degreasing, and dry cleaning [1]. The primary exposure routes in community settings are vapor inhalation from dry-cleaned fabrics [2] and contaminated soil [1], and ingestion of contaminated water [1]. The latest drinking water assessment from the US Geological Survey detected PCE in 7% of surface water samples and 24% of groundwater samples, making it a common drinking water contaminant in the United States [3].

While improper waste disposal is the typical source of PCE water contamination [1], residents of Massachusetts and Rhode Island were exposed to PCE-contaminated drinking water from 1968 through the early 1990s when local water departments installed vinyl-lined (VL) asbestos cement (AC) water mains. The vinyl liner was sprayed onto the inner pipe surface in a slurry of vinyl toluene resin and PCE to address taste and odor problems. VLAC pipes were shipped to the water departments for installation after drying for 48 h [4]. Because PCE is volatile, it was assumed that most would evaporate before the pipes were installed. However, water samples taken by state officials in 1980 revealed that PCE had persisted in the liner and had leached into the public drinking water supplies [5]. State surveys revealed that hundreds of miles of VLAC pipes had been installed throughout New England, mainly in Massachusetts (MA) and Rhode Island (RI) [6].

Because the lined pipes were used to replace existing pipe and to extend the water system as the resident population grew, the pattern of PCE contamination was irregular. Adjacent streets and even adjacent houses had different pipes and contaminant levels, leading to a natural experiment reminiscent of John Snow’s cholera investigation in 1854 London [7]. The contamination pattern also led to a wide range of exposure levels. In 1980 PCE levels in MA water monitoring samples ranged from 1.5 to 80 μg/l in medium and high-flow pipes and 1600 to 7750 μg/l in low-flow pipes [4]. Levels in RI monitoring samples from eleven systems had levels that exceeded the 1980 EPA suggested no adverse response level (SNARL) of 40 μg/L [8]. Remediation to achieve water levels below 40 μg/L was subsequently instituted mainly using bleeder valves and regular line flushing [4]. The current maximum contaminant level is 5 μg/L and reported levels in MA and RI are now below this level [9, 10].

Although PCE is considered “probably carcinogenic” to humans [11] and neurotoxic [1], its effects on reproduction, particularly fetal death, are less understood. Animal experiments suggest a harmful effect of prenatal exposure to PCE and the closely related solvent trichloroethylene (TCE) on offspring viability in rats, chicks, and rabbits [12–21]. Epidemiological studies of women with occupational exposure to dry cleaning solvents have also observed positive associations for pregnancy loss [22, 23]. However, only a few studies have examined fetal deaths among women exposed in community settings via contaminated drinking water [24–28]. Two of these studies found increases in the risk of fetal death among highly exposed women, but the results were based on a small number of cases [26, 28]. In particular, a cross-sectional study in Woburn, MA with 19 fetal deaths found that women highly exposed to well water contaminated with PCE and several other chemicals had a 2.6-fold increased risk fetal death (95% CI: 0.7–8.9) [26]. Our retrospective cohort study in Cape Cod, MA with 27 fetal deaths found that women with high levels of prenatal PCE exposure had 2.4 times the risk of stillbirth at > = 27 weeks’ gestation (95% CI: 1.0–5.6) and 1.4 times the risk of placental abruption (95% CI:0.7–2.7), a common cause of stillbirth [28]. The present case-control study was undertaken to examine further the association between PCE-contaminated drinking water and the risk of placenta-related stillbirth with a large number of cases from MA and RI.

## Methods

### Selection of study population

Cases were comprised of stillborn infants who were delivered from 1968 through 1995 to residents of 24 MA and four RI cities and towns with some VLAC water pipes. Approximately 480 miles of VLAC pipes were installed in these towns, representing approximately 63% of the VLAC pipes in the two states. The remaining RI and MA towns with VLAC pipes were excluded from the present study because they had few VLAC pipes, lacked documentation on the locations and dates of VLAC pipe installation (precluding our ability to conduct exposure assessments), and/or small resident populations. Available water sampling data indicated that PCE contamination persisted in public water supplies of selected towns through the 1990s because the target level for remediation was 40 μg/L [29].

Cases were identified by manually reviewing cause of death information recorded in approximately 1900 fetal death certificates from residents of selected cities and towns. MA certificates are required for stillborn fetuses at or after 20 weeks’ gestation and/or weighing > = 350 g, while RI certificates are required for stillbirths after 20 weeks’ gestation, irrespective of fetal weight. Because the causes of stillbirth are quite heterogeneous (including maternal diseases, umbilical cord accidents, congenital anomalies and placental disorders) [30], etiological research examining all stillbirths as a combined outcome likely results in substantial outcome misclassification. Therefore, we selected only stillbirths whose cause of death information listed “placental abruption” and/or “placental insufficiency.” These related conditions were chosen because we previously found an association between PCE exposure and placental dysfunction in our prior retrospective cohort study [28]. A total of 305 placenta-related stillbirths were identified during the ascertainment period; 301 remained after excluding four duplicate stillbirths.

Liveborn controls were randomly selected from birth records of infants born during the same time period to residents of the same MA and RI cities and towns as the cases. The control selection process was stratified by state and year so that the number of controls selected from each state was proportional to the number of births in the study towns across the long case ascertainment period (i.e. 45% for RI and 55% for MA). A total of 800 controls were targeted for selection; 794 remained after excluding duplicate subjects.

### Questionnaire and vital record data collection

Fetal death and livebirth certificates and computerized vital record data were abstracted to obtain parents’ and infants’ names; maternal address at delivery; infant’s date of birth; maternal and paternal age, race, and educational level; maternal pregnancy history; date of last menstrual period; prenatal care information and gestational age.

Mothers were also traced using Internet-based resources and sent self-administered questionnaires. Overall, 18% of case mothers and 7% of control mothers were found to be deceased. We successfully located 72 and 88% of living case and control mothers, respectively, and, of these, 35% of case mothers and 32% of control mothers returned the questionnaire after two mail and one telephone reminder. The purpose of the questionnaire was to augment vital records data on confounding variables and obtain information on water source.

When we compared the demographic characteristics of questionnaire respondents and non-respondents, we found that the two groups were similar with respect to PCE exposure status, race, educational level, and parity. While respondents were more likely to reside in Massachusetts (73% of respondents versus 49% of non-respondents), be older at delivery (median age was 28.2 for respondents and 26.7 for non-respondents), and begin prenatal care in the first trimester (90.7% for respondents and 84.6% for non-respondents), these differences were present for both case and control mothers who returned our study questionnaire.

### Geocoding of residential addresses

Residential addresses at birth as recorded in the vital records were geocoded to a latitude and longitude using ArcGIS (10.0, ESRI, Redlands, CA). Prenatal addresses of questionnaire respondents who lived at a different address during the first trimester were also geocoded (*N* = 41). Whenever possible, each address was assigned to a parcel of land. Addresses that could not be geocoded to the parcel matching the address were geocoded to the closest parcel address by street number. When the street number was unavailable (*N* = 6), the address was geocoded to the middle of the street when the street was less than a mile long and to the intersection of the address with the cross-street when the street was a mile or longer. Overall 98.5% of the addresses were successfully geocoded. All geocoding was conducted without knowledge of case/control status.

### PCE exposure assessment

Because historical water sampling data were sparse, we used a leaching and transport model to assess a woman’s PCE exposure. The model, which was developed by Webler and Brown for our epidemiological research [31, 32], estimated the mass of PCE delivered to each residence during the birth year as well as the first and second trimester year. Results from a prior validation study indicate good correlation between our modeled exposure estimates and PCE concentrations in historical water samples from Cape Cod (Spearman correlation coefficient (ρ) = 0.65, *p* < 0.00010 [33].

The mass of PCE entering the drinking water was estimated using the initial amount of PCE in the pipe liner, the pipe’s age, the leaching rate of PCE from the liner into the water, and the water flow rate through the pipe. The pipe’s initial stock of PCE was based on the pipe dimensions, and information from the manufacturers on the application of the liner. The leaching rate of PCE, which declines with time, was determined by Demond [4]. The estimate of the magnitude and direction of the water flow is a function of the configuration of the water distribution system and number of water users. We estimated water flow and direction by incorporating the Webler and Brown algorithm into the open source code of water distribution system modeling software (EPANET) developed by the US EPA [34]. Initially designed for water quality monitoring programs, EPANET software has been used in several epidemiological studies of drinking water contaminants “e.g. [35]”.

Using GIS maps of geocoded birth residences and the study towns’ water distribution systems, we created a schematic for each town depicting the locations of the water sources and pipes (with information on their length, diameter and composition), and points along the pipe indicating where water consumption occurred. We assigned each birth residence to the closest consumption point on the distribution system. The model simulated the instantaneous flow of water through each system’s pipe network and estimated the mass of PCE in grams delivered to each subject’s residence during the birth year. Because only pipe installation years were known, it was not possible to obtain monthly exposure estimates. Instead, we estimated average monthly PCE exposure level during the birth year by taking 1/12th of the annual mass of PCE that entered an exposed residence during that year. All questionnaire respondents who report using a private well for their prenatal water supply (*n* = 22) were considered unexposed. Bottled water use was rarely reported by questionnaire respondents (*n* = 24) and not considered a source of PCE exposure.

### Data analysis

The primary analysis of associations between stillbirth and PCE exposure first compared mothers who were ever exposed to PCE-contaminated drinking water during the birth year to unexposed mothers. Next, we examined four exposure levels based on the distribution of PCE exposure in control subjects. Low exposure was defined as less than or equal to the median exposure value, moderate exposure was defined as above the median exposure value through the 90th percentile and high exposure was defined as above the 90th percentile. We also dichotomized the average monthly exposure during the birth year at the level corresponding to an average drinking water concentration of 40 μg/L, the suggested action guideline for remediation when the PCE contamination was discovered. To assess the possibility of a non-linear relationship between PCE exposure and stillbirth, we also fit restricted cubic splines. Splines were truncated at the 99th percentile of PCE exposure due to the highly right-skewed distribution. Lastly, we conducted sensitivity analyses to examine exposure during the calendar year of the first and second trimesters and take into account respondents who reported moving during the pregnancy. The referent group for all analyses was always comprised of unexposed mothers.

The strength of the association between PCE exposure and the occurrence of stillbirth was estimated with odds ratios (OR) and statistical stability was evaluated with 95% confidence intervals (CI). We examined all stillbirths combined and stillbirths due to placental abruption and placental insufficiency separately. Cases who had both conditions listed on the fetal death certificate contributed to each subgroup.

Multiple logistic regression models were used to estimate ORs while controlling for confounding variables. We selected potential confounders from those available in the vital records and self-administered questionnaires based on a literature review and construction of a directed acyclic graph; see Table 1 for variables that were considered. Multiple imputation was used to obtain values of potential confounders with missing data. Missingness for potential confounders ranged from 0% (e.g., state, delivery year) to 76% (e.g., maternal occupational exposure to solvents). Twenty imputed data sets were generated using the fully conditional specification (FCS) multiple imputation method based on 27 variables, including PCE exposure and case/control status. Point and variance estimates from the imputed data sets were subsequently combined and used in adjusted analyses. State of birth (MA, RI), delivery year (1968–1978, 1979–1988, 1989–1995) and prenatal exposure to smoking (yes, no) were included in the final logistic regression models based on a priori considerations. Additional variables that changed the crude association between stillbirths and PCE exposure by > = 5% were also included in the final models; these variables were paternal educational level (less than high school, high school graduate, some college, college graduate) and receipt of prenatal care during the first trimester (yes, no). Even though many remaining variables were associated with case-control status, they were not associated with the exposure and, therefore, did not confound the associations under study. All statistical analyses were conducted using SAS 9.4 (SAS Institute, Cary North Carolina).

**Table 1:** Distribution of Selected Characteristics of Cases and Controls

Characteristic	Stillbirth Cases		Controls	
	(*N* = 296)		(*N* = 783)	
	n	%	n	%
State of Birth				
Massachusetts	155	52.4	442	56.4
Rhode Island	141	47.6	341	43.6
Year of delivery				
1968–1978	165	55.7	278	35.5
1979–1988	79	26.7	293	37.4
1989–1995	52	17.6	212	27.1
Maternal age at delivery (mean, sd)	27.0 (5.9)		27.0 (5.5)	
Missing	1		0	
Paternal age at delivery (mean, sd)	30.1 (6.8)		29.8 (6.1)	
Missing	77		34	
Maternal Race				
White	153	91.6	623	90.0
Non-white	14	8.4	69	10.0
Missing	129		91	
Maternal Educational Level				
Less than high school	19	12.8	96	14.1
High school graduate	57	38.3	247	36.2
Some college	28	18.8	175	25.7
College graduate	45	30.2	164	24.0
Missing	147		101	
Paternal Educational Level				
Less than high school	23	16.3	90	13.8
High school graduate	48	34.0	221	33.9
Some college	19	13.5	139	21.3
College graduate	51	36.2	202	31.0
Missing	155		131	
Prenatal care began in first trimester				
Yes	94	81.7	485	88.2
No	21	18.3	65	11.8
Missing	181		233	
Prior livebirths				
Yes	105	62.5	388	56.4
No	63	37.5	300	43.6
Missing	128		95	
Prior pregnancy losses				
Yes	19	24.4	70	12.9
No	59	75.6	473	87.1
Missing	218		240	
Prenatal smoking				
Yes	18	31.0	70	23.3
No	40	69.0	230	76.7
Missing	238		483	
Prenatal alcoholic beverage consumption				
Yes	17	29.8	72	35.0
No	40	70.2	134	65.0
Missing	239		577	
Maternal occupational exposure to solvents				
Yes	2	3.4	4	2.0
No	57	96.6	197	98.0
Missing	237		582	

## Results

The current analysis includes 296 cases and 783 controls. Five cases and 11 controls were excluded because their residential information was insufficient for geocoding. As shown in Table 1, demographic characteristics of case and control mothers, including state of residence, age at delivery, and race were similar. However, there were several differences between the groups, including a higher proportion of case mothers (and their partners) who were college graduates, received prenatal care during the first trimester, had prior livebirths, prior pregnancy losses, and smoked cigarettes. A higher proportion of case vs. control mothers delivered during the early years of the exposure period but fewer case mothers consumed alcoholic beverages during pregnancy.

Overall, 75.7% of case and 69.6% of control mothers had some PCE exposure during the birth year. Both exposed case and control mothers had a wide range of exposure levels spanning several orders of magnitude but exposure levels were higher among exposed case mothers (Table 2). For example, the median level was 9.99 × 10^− 3^ g among exposed case mothers versus 5.40 × 10^− 3^ g among exposed control mothers. Mothers with any PCE exposure during the delivery year had a 1.4-fold increase in the crude odds of stillbirth (95% CI: 1.0–1.8) that rose to 1.7 (95% CI: 1.2–2.4) when confounders were controlled (Table 3). Adjusted odds ratios increased in a dose-dependent fashion from 1.5 (95% CI: 1.0–2.3) for low exposure (> 0-median) to 1.7 (95% CI: 1.1–2.5) for moderate exposure (>median-90th percentile) and 1.9 (95% CI: 1.1–3.2) for high exposure (>90th percentile) (*p* value for trend 0.02). A similar pattern was observed when PCE exposure was dichotomized at 40 μg/L. Adjusted odds ratios were 1.5 (95% CI: 1.1–2.2) and 2.6 (95% CI: 1.4–4.8), respectively, for PCE exposure up to and over the 40 μg/L action level (p value for trend = 0.003). The results of the restricted cubic spline regression supported a linear dose-response relationship.

**Table 2:** Distribution of Average Monthly PCE Exposure (g) During Birth Year Among Exposed Case and Control Mothers

	Exposed Case Mothers	Exposed Control Mothers
Minimum	4.14 × 10^−6^	2.08 × 10^−8^
10th Percentile	1.47 × 10^−4^	5.00 × 10^−5^
25th Percentile	8.26 × 10^−4^	5.18 × 10^−4^
Median	9.99 × 10^−3^	5.40 × 10^−3^
75th Percentile	7.84 × 10^−2^	5.57 × 10^−2^
90th Percentile	9.81 × 10^−1^	4.56 × 10^− 1^
Maximum	40.5	81.81

**Table 3:** Frequencies, Odds Ratios and 95% Confidence Intervals for Stillbirth According to Average Monthly PCE Exposure (in grams) During Birth Year

	Number of Cases	Number of Controls	Crude Odds Ratio (95% CI)	Multivariate^a^ Odds Ratio (95% CI)
*All Stillbirths* ^*b*^				
Any Exposure	224	545	1.4 (1.0–1.8)	1.7 (1.2–2.4)
No Exposure	72	238	1.0 (---)	1.0 (---)
Exposure Categorized in Percentiles^c^				
> = 90th	32	55	1.9 (1.2–3.2)	1.9 (1.1–3.2)
> 50th - < 90th	95	217	1.4 (1.0–2.1)	1.7 (1.1–2.5)
> 0 - 50th	97	273	1.2 (0.8–1.7)	1.5 (1.0–2.3)
0 (Referent)	72	238	1.0 (---)	1.0 (---)
Exposure Categorized by 1980 Action Level^d^				
> 40 μg/L	25	30	2.8 (1.5–5.0)	2.6 (1.4–4.8)
> 0 - < =40 μg/L	199	515	1.3 (0.9–1.7)	1.5 (1.1–2.2)
0 (Referent)	72	238	1.0 (---)	1.0 (---)
*Stillbirths due to Placental Abruption*				
Any Exposure	159	545	1.4 (1.0–2.1)	1.7 (1.1–2.6)
No Exposure	48	238	1.0 (---)	1.0 (---)
Exposure Categorized in Percentiles^c^				
> = 90th	21	55	1.9 (1.0–3.4)	1.9 (1.0–3.5)
> 50th - < 90th	65	217	1.5 (1.0–2.3)	1.7 (1.1–2.7)
> 0 - 50th	73	273	1.3 (0.9–2.0)	1.6 (1.0–2.6)
0 (Referent)	48	238	1.0 (---)	1.0 (---)
Exposure Categorized by 1980 Action Level^d^				
> 40 μg/L	16	30	2.6 (1.3–5.2)	2.5 (1.3–5.2)
> 0 - < =40 μg/L	143	515	1.4 (1.0–2.0)	1.6 (1.1–2.4)
0 (Referent)	48	238	1.0 (---)	1.0 (---)
*Stillbirths Due to Placental Insufficiency*				
Any Exposure	73	545	1.3 (0.8–2.1)	1.7 (1.0–3.0)
No Exposure	25	238	1.0 (---)	1.0 (---)
Exposure Categorized in Percentiles^c^				
> = 90th	12	55	2.1 (1.0–4.4)	1.9 (0.9–4.2)
> 50th - < 90th	35	217	1.5 (0.9–2.6)	1.9 (1.0–3.5)
> 0 - 50th	26	273	0.9 (0.5–1.6)	1.4 (0.7–2.9)
0 (Referent)	25	238	1.0 (---)	1.0 (---)
Exposure Categorized by 1980 Action Level^d^				
> 40 μg/L	10	30	3.2 (1.4–7.2)	2.7 (1.2–6.5)
> 0 - < =40 μg/L	63	515	1.2 (0.7–1.9)	1.6 (0.9–2.8)
0 (Referent)	25	238	1.0 (---)	1.0 (---)

^a^Controlled for state of birth, delivery year, paternal educational level, receipt of prenatal care during the first trimester

^b^Nine stillbirth cases had both placental abruption and placental insufficiency

^c^Cut points for the 50th and 90th percentiles were 5.4 × 10^− 3^ and 4.6 × 10^− 1^ g, respectively

^d^Cut point for 40 μg/l was 1.14 g

No meaningful changes in these findings were observed in sensitivity analyses using first and second trimester exposure levels or accounting for maternal changes in residence (data not shown). Similar dose-dependent increases in odds ratios were present for stillbirths due to placental abruption and placental insufficiency (Table 3).

## Discussion

The present study has found evidence that pregnant women exposed to PCE-contaminated drinking water have a dose-dependent increase in the odds of stillbirth due to placental abruption and placental insufficiency. These findings are consistent with animal experiments suggesting that PCE and TCE cause species-specific increases in embryotoxicity. Increased rates of resorbed implants and fetuses have been observed in studies of pregnant rats, Leghorn chickens, and rabbits but not mice [12–16, 18–21]. These findings are supported by experimental evidence in pregnant mice that PCE and its metabolite trichloroacetic acid accumulate in amniotic fluid and fetal tissues following inhalation exposure [36].

Numerous occupational studies with specific exposure definitions, such as dry cleaning work, have observed an increased risk of pregnancy loss “e.g., [37–43]”. Other occupational studies also have found positive associations between maternal occupational exposure to solvent mixtures and the risk of pregnancy loss “e.g., [22, 23]”. However, results are difficult to interpret because many types of solvents and jobs were included in the exposed group.

Previous reports of pregnancy loss following exposure to contaminated drinking water have mixed results. In New Jersey, no association was reported in a cross-sectional study of town-level PCE or trichloroethylene (TCE) and fetal loss occurring at > = 20 weeks’ gestation where maximum monthly exposure levels were 55 ppb for TCE and 26 ppb for PCE [25]. Another cross-sectional study of Woburn, MA residents also found no increase in the risk of spontaneous abortion among women exposed to well water contaminated with PCE (21 ppb), TCE (276 ppb) and several other chemicals (e.g. chloroform, 12 ppb) [24]. Woburn study investigators obtained data on spontaneous abortions from subject interviews, and estimated prenatal exposure to the contaminated wells using a water distribution model. Stillbirths were not examined as a separate outcome in this study.

In contrast, a follow-up study in Woburn found a 1.8-fold increased risk of fetal death at > = 20 weeks’ gestation among residents with any exposure during pregnancy (95% CI: 0.4–6.6), and a 2.6-fold increased risk of fetal deaths (95% CI: 0.7–8.9) among women highly exposed during pregnancy (>90th percentile) [26, 44]. Vital record reports of fetal deaths and a more sensitive exposure assessment model were used in the follow-up study.

Our retrospective cohort study from Cape Cod MA examined associations of prenatal PCE exposure from the VLAC pipes and pregnancy loss. Initial analyses found no association for first trimester loss and second and third trimester loss combined [27]; however, a subsequent analysis focused on placental dysfunction disorders found that pregnancies with PCE exposure > = the median had 2.4 times the risk of stillbirth at > = 27 weeks’ gestation (95% CI: 1.0–5.6) and 1.4 times the risk of placental abruption (95% CI: 0.7–2.7) [28]. Both the Woburn and Cape Cod study results were statistically unstable because analyses were based on 19 and 27 fetal deaths, respectively. Only two stillbirth cases in our prior cohort analysis of placental dysfunction disorders were selected for the current case-control study; the small degree of overlap between the two studies was likely due to design differences, including geographic area, time period and method of case ascertainment. Exclusion of these two cases did not alter the results.

The current study has numerous strengths, including a relatively large sample size, wide range of exposure levels, and lack of recall bias through the use of vital records for identifying stillbirth cases and independent exposure assessments. However, the findings are likely affected by exposure misclassification. Because historical exposure measurements were unavailable, we estimated the mass of PCE delivered to each residence during the birth year using an exposure model with numerous assumptions [31]. Furthermore, a relatively small proportion of mothers completed questionnaire on water consumption and bathing habits during pregnancy, and so we could not incorporate behavioral data into our exposure assessments. However, a previous analysis that included this information in the PCE model did not meaningfully change subjects’ exposure classification [45].

Even though results from a prior validation study indicate good correlation between our modeled exposure estimates and PCE concentrations in historical water samples from Cape Cod (Spearman correlation coefficient (ρ) = 0.65, *p* < 0.00010 [33], non-differential exposure misclassification likely attenuated the associations observed in the highest category. We were unable to identify a sufficient number of reliable historical water samples from other communities in the present study and so could not conduct a validation study in a wider geographic area.

Our primary analysis examined PCE exposure during the birth year because we considered it the most relevant exposure window for the stillbirths under study. Nevertheless, the results were unchanged in sensitivity analyses using either first or second trimester exposure windows and accounting for changes in maternal residence during pregnancy. This was not surprising given that PCE exposure level during the birth year was highly correlated with that of the first and second trimesters (Pearson correlation coefficients comparing 0.94 and 0.95 respectively, *p* < 0.001) and that a small number of mothers reported moving during pregnancy.

Since VLAC pipes were used to replace existing pipes and extend the water distribution system to accommodate population growth, the exposure distribution was irregular. Consequently, there was little confounding even though several variables were associated with case-control status. The low questionnaire response rate necessitated the use of multiple imputation procedures to fill in missing values on confounders; however, these procedures are considered the most valid method for handling this problem [46]. Nevertheless, we cannot rule out the possibility that residual confounding due to unmeasured or poorly measured confounding variables impacted the results.

Selection bias was unlikely during subject ascertainment because we were unaware of a woman’s exposure status when we identified the cases and controls. While the questionnaire response rate was low, women who chose not to respond to our survey were similar to women who did respond with respect to PCE exposure status, race, and parity. Non-respondents were more likely to reside in Rhode Island and be younger at delivery, and less likely to have a college education and begin prenatal care in the first trimester. These differences were present for non-respondent cases *and* controls and so should not have biased the questionnaire data. Furthermore, the associations between PCE exposure and stillbirth were examined in the entire study population, irrespective of respondent status.

Because we investigated stillbirths occurring at 20 weeks’ gestation or later, it is possible that a differential loss of pregnancies before this point may have led us to underestimate the true effect of PCE exposure. Such a ‘competing mortality’ bias could exist if the pregnancies most susceptible to PCE-induced stillbirths were not included in our analysis because they were more likely to be spontaneously aborted earlier in pregnancy. Previous findings from this cohort, however, suggest that prenatal PCE exposure is not associated with early pregnancy loss [27].

In summary, we observed a dose-dependent increase in the odds of stillbirth due to placental abruption and placental insufficiency among pregnant women exposed to PCE contaminated drinking water. While PCE contamination from old VLAC pipes is no longer a problem in Massachusetts and Rhode Island (10, 29), the solvent continues to be a common contaminant of public drinking water supplies in the U.S. because of improperly managed waste disposal [1]. Thus, these findings highlight the importance of considering pregnant women and their developing fetuses when monitoring, regulating, and remediating drinking water supplies.

## Abbreviations

CI: Confidence interval
GIS: Geographic information systems
OR: Odds ratio
PCE: Tetrachloroethylene
SNARL: Suggested no adverse response action level
TCE: Trichloroethylene
VLAC: Vinyl-lined asbestos-cement

## References

[1] U.S. Environmental Protection Agency. Toxicological review of tetrachloroethylene (perchloroethylene). Washington, DC: US Environmental Protection Agency; 2012.

[2] Sherlach KS, Gorka AP, Dantzler A, Roepe PD (2011). Quantification on perchloroethylene residues in dry-cleaned fabrics. Environ Tox Chem.

[3] Focazio MJ, Kolpin DW, Barnes KK, Furlong ET, Meyer MT, Zaugg SD (2008). A national reconnaissance for pharmaceuticals and other organic waterwater contaminants in the United States –II untreated drinking water sources. Sci Total Environ.

[4] Demond AH. A source of tetrachloroethylene in the drinking water of New England: an evaluation of toxicity of tetrachloroethylene and the prediction of its leaching rates from vinyl-lined asbestos-cement pipe. MS thesis, Massachusetts institute of technology; 1982.

[5] Massachusetts Department of Environmental Quality Engineering (MDEQE) (1982). Status report on tetrachloroethylene contamination of public drinking water supplies by vinyl-lined asbestos cement pipe.

[6] Larson CD, Love T, Reynolds G (1983). Tetrachloroethylene leached from lined asbestos-cement pipe into drinking water. JAWWA.

[7] Snow J. Snow on Cholera. A reprint of two papers of John Snow, MD together with a biographical memoir by B.W. Richardson, MD and an Introduction by Wade Hampton Frost, MD. Facsimile of 1936 Edition. New York: Hafner Publishing Co. p. 1965.

[8] State of Rhode Island and Providence Plantations. Inter-Office Memo: Asbestos cement (A.C.) vinyl-lined pipe preliminary survey for tetrachloroethylene determination; October 6, 1980.

[9] Massachusetts Department of Environmental Protection. Tetrachloroethylene Sampling Results. 2013.

[10] Rhode Island Department of Health. Center for Drinking Water Quality. 2016 Annual Report. Providence, RI: Rhode Island Department of Health, 2016. http://www.health.ri.gov/publications/annualreports/2016DrinkingWaterQualityCompliance.pdf. Accessed 6 Feb 2018.

[11] International Agency for Research on Cancer (2014). IARC Monographs on the evaluation of carcinogenic risks to humans, vol 106. Some chlorinated solvents and their metabolites.

[12] Healy TEJ, Poole TR, Hopper A (1982). Rat fetal development and maternal exposure to trichloroethylene 100 ppm. Br J Anaesth.

[13] Hardin B, Bond GP, Sikov MR, Andrew FD, Beliles RP, Niemeier RW (1981). Testing of selected workplace chemicals for teratogenic potential. Scand J Work Environ Health.

[14] Belilies RP, Brusick DJ, Mecler FJ. Teratogenic-mutagenic risk of workplace contaminants: trichloroethylene, perchloroethylene, and carbon disulfide. NIOSH Contract Report No. 210–77-0047. NTIS Publ. No. PB-82-185-075. Springfield, VA: National Technical Information Service; 1980.

[15] Bross G, Di Franceisco D, Desmond ME (1983). The effects of low dosages of trichloroethylene on chick development. Toxicology.

[16] Elovaara E, Hemminki K, Vainio H (1979). Effects of methylene chloride, trichloroethane, trichloroethylene, tetrachloroethylene, and toluene on the development of chick embryos. Toxicology.

[17] Nelson BK, Taylor BJ, Setzer JV, Hornung RW (1980). Behavioral toxicology of perchloroethyne in rats. J Environ Path Toxicol.

[18] Schwetz BA, Leong BKJ, Gehring PJ (1975). The effect of maternally inhaled trichloroethylene, perchloroethylene, methyl chloroform, and methylene chloride on embryonal and fetal development in mice and rats. Toxicol Appl Pharmacol.

[19] Smith MK, Randall JL, Read EJ, Stober JA (1989). Teratogenic activity of trichloroacetic acid in the rat. Teratology.

[20] Tinston DJ (1995). Perchloroethylene: multigenerational inhalation study in the rat. Report no. CTL/P/4097. Sponsored by the Halogenated Solvents Industry Alliance, HSIA/90/0002.

[21] Narotsky MG, Kavlock RJ (1995). A multidisciplinary approach to toxicological screening: II developmental toxicity. J Toxicol Environ Health.

[22] Khattak S, Moghtader GK, McMartin K, Barrera M, Kennedy D, Koren G (1999). Pregnancy outcome following gestational exposure to organic solvents: a prospective controlled study. JAMA.

[23] Attarchi MA, Ashouri M, Labbafinejad Y, Mohammadi S (2012). Assessment of time to pregnancy and spontaneous abortion status following occupational exposure to organic solvents mixture. Int Arch Occup Environ Health.

[24] Lagakos SW, Wessen BJ, Zelen M (1986). An analysis of contaminated well water and health effects in Woburn, Massachusetts. J Am Stat Assoc.

[25] Bove FJ, Fulcomer MC, Klotz JB, Esmart J, Dufficy EM, Savrin JE (1995). Public drinking water contamination and birth outcomes. Am J Epidemiol.

[26] Massachusetts Department of Public Health (1996). The Woburn environment and birth study synopsis. Boston: Massachusetts department of Public Health.

[27] Aschengrau A, Weinberg J, Gallagher L, Winter M, Vieira V, Webster T (2009). Prenatal exposure to tetrachloroethylene-contaminated drinking water and the risk of pregnancy loss. Water Qual Expo Health.

[28] Carwile JL, Winter M, Mahalingaiah S, Aschengrau A (2014). Prenatal Drinking-Water Exposure to Tetrachloroethylene and Placental Dysfunction Disorders. Environ Health.

[29] Massachusetts Department of Environmental Protection (2013). Tetrachloroethylene sampling results database.

[30] Stillbirth Collaborative Research Network Writing Group (2011). Causes of death among stillbirths. JAMA.

[31] Webler T, Brown HS (1993). Exposure to tetrachloroethylene via contaminated drinking water pipes in Massachusetts: a predictive model. Arch Environ Health.

[32] Aschengrau A, Ozonoff D, Paulu C, Coogan P, Vezina R, Heeren T (1993). Cancer risk and Tetrachloroethyene-contaminated drinking water in Massachusetts. Arch Environ Health.

[33] Gallagher LG, Vieira VM, Ozonoff D, Webster TF, Aschengrau A (2011). Risk of breast cancer following exposure to tetrachloroethylene-contaminated drinking water in cape cod, Massachusetts: reanalysis using a modified exposure assessment. Environ Health.

[34] U.S. Environmental Protection Agency. EPANET 2.0. https://www.epa.gov/water-research/epanet. Accessed 14 Nov 2017.

[35] Aral MM, Maslia ML, Ulirsch GV, Reyes JJ (1996). Estimating exposure to volatile organic compounds from municipal water-supply systems: use of a better computational model. Arch Environ Health.

[36] Ghantous H, Danielsson BRG, Dencker L, Gorczak J, Vesterberg O (1986). Trichloroacetic acid accumulates in murine amniotic fluid after tri-and tetrachloroethylene inhalation. Acta Pharmacol et toxicol.

[37] Bosco MG, Figa-Talamanca I, Salerno S (1987). Health and reproductive status of female workers in dry cleaning shops. Int Arch Occup Environ Health.

[38] Kyyronen P, Taskinen H, Lindbohm ML, Hemminki K, Heinonen OP (1989). Spontaneous abortions and congenital malformations among women exposed to tetrachloroethylene in dry cleaning. J Epidemiol Comm Health.

[39] Kolstad HA, Brandt LPA, Rasmussen KC (1990). Klorerede oplosningsmidler og fosterskader. Ogeskr Laeger.

[40] Olsen J, Hemminki K, Ahlborg G, Bjerkedal T, Kyyronen P, Taskinen H (1990). Low birthweight, congenital malformations, and spontaneous abortions among dry-cleaning workers in Scandinavia. Scand J Work Environ Health.

[41] Lindbohm MJ, Taskinen H, Sallmen M, Hemminki K (1990). Spontaneous abortion among women exposed to organic solvents. Am J Indust Med.

[42] Windham GC, Shusterman D, Swan SH, Fenster L, Eskenazi B (1991). Exposure to organic solvents and adverse pregnancy outcome. Am J Ind Med.

[43] Doyle P, Roman E, Beral V, Brookes M (1997). Spontaneous abortion in dry cleaning workers potentially exposed to perchloroethylene. Occup Environ Med.

[44] Bove F, Shim Y, Zeitz P (2002). Drinking water contaminants and adverse pregnancy outcomes: a review. Environ Health Perspect.

[45] Vieira V, Aschengrau A, Ozonoff D (2005). Impact of tetrachloroethylene-contaminated drinking water on the risk of breast cancer: using a dose model to assess exposure in a case-control study. Environ Health.

[46] Rothman KJ, Greenland S, Lash TL (2008). Modern Epidemiology, 3rd Edition.

